# The Neurotensin Receptor-1 Pathway Contributes to Human Ductal Breast Cancer Progression

**DOI:** 10.1371/journal.pone.0004223

**Published:** 2009-01-19

**Authors:** Sandra Dupouy, Véronique Viardot-Foucault, Marco Alifano, Frédérique Souazé, Geneviève Plu-Bureau, Marc Chaouat, Anne Lavaur, Danielle Hugol, Christian Gespach, Anne Gompel, Patricia Forgez

**Affiliations:** 1 INSERM-UPMC CDR Saint-Antoine EQ.5, Hôpital Saint-Antoine, Paris, France; 2 Unité de Gynécologie, Université Paris Descartes, AP-HP, Hôtel-Dieu de Paris, Paris, France; 3 Service de chirurgie thoracique, Université Paris Descartes, AP-HP, Hôtel-Dieu de Paris, Paris, France; 4 Service de chirurgie plastique, AP-HP, Hôpital Rotschild, Bd Picpus, Paris, France; 5 Département d'Anatomo-pathologie, Université Paris Descartes, AP-HP, Hôtel-Dieu de Paris, Paris, France; Health Canada, Canada

## Abstract

**Background:**

The neurotensin (NTS) and its specific high affinity G protein coupled receptor, the NT1 receptor (NTSR1), are considered to be a good candidate for one of the factors implicated in neoplastic progression. In breast cancer cells, functionally expressed NT1 receptor coordinates a series of transforming functions including cellular migration and invasion.

**Methods and Results:**

we investigated the expression of NTS and NTSR1 in normal human breast tissue and in invasive ductal breast carcinomas (IDCs) by immunohistochemistry and RT-PCR. NTS is expressed and up-regulated by estrogen in normal epithelial breast cells. NTS is also found expressed in the ductal and invasive components of IDCs. The high expression of NTSR1 is associated with the SBR grade, the size of the tumor, and the number of metastatic lymph nodes. Furthermore, the NTSR1 high expression is an independent factor of prognosis associated with the death of patients.

**Conclusion:**

these data support the activation of neurotensinergic deleterious pathways in breast cancer progression.

## Introduction

Breast cancer is the most frequent cause of cancer-related deaths among women in the western world [1]. Among these patients, one of four women dies from breast cancer, despite improvements in diagnosis, surgery, chemotherapy and the new targeted therapies. Death is associated with the metastatic development of the disease. The discovery and characterization of new contributors remain necessary in order to develop appropriate and highly specific treatments targeted to metastasis initiation and progression processes.

Neurotensin (NTS) is a 13 amino acids peptide formed from a large precursor, cleaved by convertases. NTS is commonly known for its distribution along the gastrointestinal tract [2]. Typical physiological functions for NTS include stimulation of pancreatic and biliary secretions, inhibition of small bowel and gastric motility, and facilitation of fatty acids translocation [3]–[5]. NTS was equally reported in functions linked specifically to neoplastic progression, including proliferation of the pancreas, prostate, colon, and lung cancer cells [6]. We have previously described detrimental effects, caused by NTS, on xenografted breast tumor growth as well as migration, invasion, and survival of breast cancer cells [7], [8].

NTS expression is also found in endocrine tumors and is linked to tissue differentiation [9]. NTS is expressed in fetal colon, repressed in newborn and adult colon, and re-expressed in approximately 25% of human colon cancers due to epigenetic mechanisms linked to NTS gene hypomethylation [10].

NTS peripheral functions are mediated through its interaction with the NTSR1 [11]. When NTSR1 is challenged with NTS, phosphatidyl inositols are hydrolyzed leading to Ca^2+^ mobilization and PKC, ERK1/2, RhoGTPases, NFkappa-B, and focal adhesion kinase (FAK) activation [12]–[15]. The NTSR1 gene is a target of the Wnt/APC oncogenic pathways connected with the β-catenin/Tcf transcriptional complex, known to activate genes involved in cancer cell proliferation and transformation [7].

In this report, we investigate the expression of NTS and NTSR1 in a cohort of 106 women diagnosed for invasive ductal breast cancer (IDCs). We conclude that NTSR1 regulation may occur in breast cancer and participates in the neoplastic progression in up to 35% of all patients.

## Results

### NTS expression in normal epithelial breast cells is regulated by estradiol

**Figure 1:**
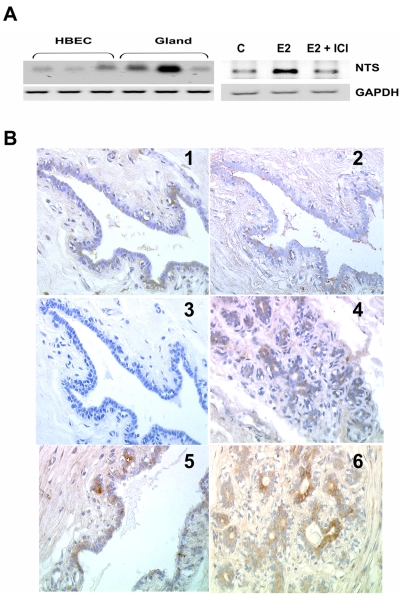
Neurotensin expression in normal breast tissue. a) *Left*, one µg of total RNA from HBEC or whole gland were reverse-transcribed and a PCR experiment specific for NTS was performed. *Right*, *o*ne µg of total RNA from HBEC cells (control, treated with 10 nM estradiol (E2) with or without 1 µM ICI 182780) was reverse-transcribed. A PCR experiment was performed using specific primers for NTS and GAPDH. b) Normal duct exposed to NTS antibody at 1/500 dilution (1), after pre-incubation with the antigen peptide for 2 h at 10 nM (2), or without primary antibody (3), and lobule exposed to NTS antibody (4). Normal tissue exposed to NTS antibody at 1/500 dilution adjacent to tumor duct (5), lobule (6). The original magnification was 200×.

The NTS gene was previously described as an estradiol target gene [16] with estradiol increasing NTS transcription in the preoptic area and neurosecretory cells of the hypothalamic arcuate nucleus [17], [18]. We hypothesized that NTS is also expressed in normal human breast tissue, and studied NTS transcript on normal mammary glands, and on eight different human breast epithelial cells (HBEC) cultures. We consistently detected NTS amplicon with low to medium intensity. Typical examples are shown in figure 1A *left*. In order to evaluate if NTS gene is also regulated by estradiol in human breast, HBEC were exposed to estradiol. As shown in figure 1A *right*, an enhancement of NTS transcripts was observed. This effect was abolished when ICI 182780, a pure anti-estrogen, was added concomitantly to estradiol (Figure 1A *right*) suggesting that estrogen receptors participate in the NTS gene regulation in human breast tissue. Corroborating these results, NTS expression was positively detected by immunohistochemistry in 19 (76%) biopsies of normal breast tissues from 25 premenopausal women. We observed NTS labeling within sparse epithelial cells of ducts and lobules (Figure 1B, 1 and 4). On the same slide we noticed that the lobular structures were labeled with a more intense staining than the duct structures. We also noticed that the normal adjacent tissue of invasive ductal breast carcinomas (IDCs) was very often labeled by NTS antibody, with similar intensity and cellular distribution as in the tissue from healthy women (Figure 1B, 5 and 6).

### NTS expression in invasive ductal breast carcinomas

**Figure 2:**
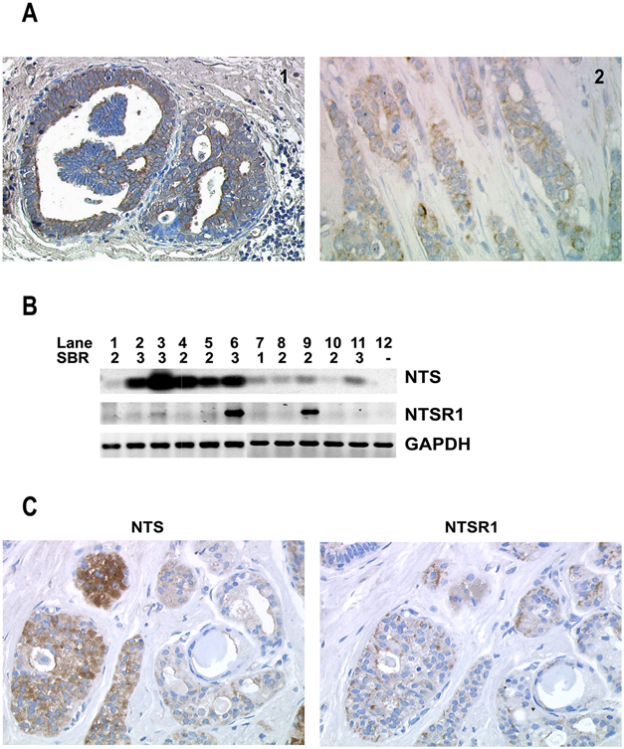
Neurotensin expression in IDCs. a) NTS immunohistochemistry was performed on IDCs, ductal (1) and invasive (2) components, magnification 200× for (1) and 400× for (2). b) NTS and NTSR1 transcripts in IDCs. One µg of total RNA from 11 patients with IDCs were reverse-transcribed, and specific PCR was performed for NTS, NT-1 receptor, or GAPDH (control). The SBR grade is indicated below each line. c) Example of NTS and NTSR1 regional co-localization by immunohistochemistry for NTS (right) and NTSR1 (left) at the original magnification 400×.

We previously demonstrated the presence of NTSR1 and NTS expression in breast ductal carcinomas, along with NTS induced effects on tumor growth, cellular mobility and collagen invasion of a cancer mammary cell line [7]. In order to further evaluate the status of NTS and NTSR1 in breast cancer we studied their respective expression in 106 IDCs. Details of clinical data, pathological characteristics, and treatment modulations are shown in table 1. NTS was graded in the invasive and ductal components in the patients' IDCs. In most cases a large amount of cells were positively labeled with NTS antibody (Figure 2A). NTS positive labeling in invasive component is significantly correlated with the positive labeling in the ductal component (P = 0.004). In both cases, NTS labeling was cytosolic (Figure 2A). Using RT-PCR, we confirmed the high expression of NTS transcript in 9 of 11 breast cancer tissues (Figure 2B). Five patients exhibited a very strong expression of NTS transcript (Figure 2B, lane 2–6) and four others displayed a weaker expression (Figure 2B, lane 7–9 and 11). No correlation was observed with prognosis factors and disease progression (tumor size, grade, number of invaded nodes, recurrence, and death) with NTS expression, neither in the ductal nor in the invasive components (Table 2). The only correlation found, was between PR and NTS expression in the invasive component. NTS is neither a marker nor associated with tumor progression in breast cancer.

**Table 1:** Patients clinical characteristics.

	IDCs
	n = 106
Age in years [mean±SD]	57.96±14.04
**Menopausal status**	
Post menopausal patients [n (% of patients)]	69 (65%)
Age at menopause [mean±SD]	50.96±3.05
Family history of breast cancer [n/number of cases studied (% of patients)]	18/79 (23%)
HRT use [n/number of cases studied (% of patients)]	17/92 (18%)
Follow-up in months [mean±SD]	67.9±41.93
Positive estrogen receptor [n/number of cases studied (% of patients)]	69/100 (69%)
Positive progesterone receptor [n/number of cases studied (% of patients)]	71/99 (71.7%)
Tumor size (cm) [mean±SD]	2.3±1.43
**SBR grade** [n]	
1	33
2	49
3	24
Number of patients N+ [n/number of cases studied (% of patients)]	42/104 (40.4%)
Number of invaded nodes [mean±SD]	1.32±2.55
Recurrence during follow-up [n (% of patients)]	26 (25%)
Deaths during follow-up [n (% of patients)]	11 (10.4%)
**Adjuvant therapy** (number of cases studied)	102
Radiotherapy [n (% of patients)]	96 (94%)
Chemotherapy [n (% of patients)]	36 (35.5%)
Tamoxifen use [n (% of patients)]	78 (76.4%)

SBR; Scarff Bloom and Richardson, n = number of patients, SD = standard deviation.

**Table 2:** Prognosis factors and deaths stratified by NT expression in the ductal and invasive components of IDCs.

	NT			NT		
	Ductal component			Invasive component		
	n = 87			n = 103		
	NO	YES	P	NO	YES	P
	n = 31	n = 56		n = 55	n = 48	
Positive estrogen receptor [n/number of cases studied (SD)]				30/49	36/47	NS
Positive progesterone receptor [n/number of cases studied (SD)]				32/51	37/45	0.034
Tumor size (cm) [number of cases studied]	30	52		54	44	
[mean±SD]	2.36±1.7	2.22±1.25	NS	2.43±1.7	2.03±0.9	NS
**SBR grade** [number of cases studied]	31	56		52	47	
1	7	21		16	17	
2	13	26		25	22	
3	11	9	NS	14	9	NS
Number of Invaded nodes [number of cases studied]	30	54		52	48	
[mean±SD]	1.17±2.3	1.66±3	NS	1.23±2	1.49±3	NS
Recurrence during follow-up [n/n of patient studied]	6/31	14/56	NS	15/55	11/47	NS
Deaths during follow-up [n/n of patients studied]	2/31	7/56	NS	5/55	6/48	NS
**Adjuvant therapy**	30	53		51	47	
Radiotherapy [n]	29	50	NS	48	46	NS
Chemotherapy [n]	12	19	NS	19	16	NS
Tamoxifen use [n]	25	38	NS	41	34	NS

### NTSR1 expression in IDCs

NTSR1 staining in IDCs showed that NTSR1 expression was spread throughout many tumor cells in the invasive and ductal components. The labeling was granular and mostly cytosolic. In the invasive component of studied IDCs, the majority exhibited a high proportion of NTSR1 positive cells (from 50 to 100%). We hypothesized that the deleterious effects of NTS previously described should occur in tumors containing a very high proportion of NTSR1 expressing cells [7], [19]. We focused on the 35% of patients in which 80% or more of the tumor cells expressed the NT1 receptor. Expression of NTSR1 was verified by RT-PCR on frozen tissues from 11 patients. As shown in Figure 2B, three patients expressed NTSR1 (lane 3, 6, 9) with two showing a very high amplicon amount (Figure 2B lane 6, 9).

**Figure 3:**
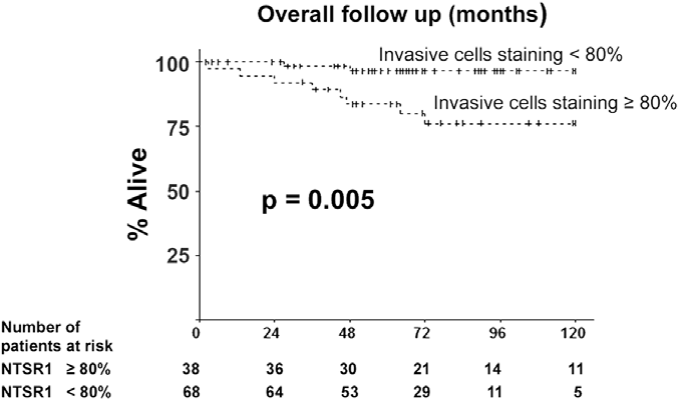
NTSR1 expression in IDCs and global survival duration. Kaplan-Maier analysis for global survival duration in both groups with low (<80%) and high (≥80%) NTSR1 expression. Probability of overall death for patients with high NTSR1 expression (n = 38) versus patients with low NTSR1 expression (n = 68).

The characteristics of the women exhibiting high NTSR1 expression (≥80% of tumor cells) are shown in table 3. High NTSR1 expression was associated with a larger tumor size (p<0.01), SBR grade 3 (p<0.05), the number of positive lymph nodes (p<0.05), and as a consequence it was also associated with chemotherapy (p<0.01). Using univariate analysis we found that patients with high expression of NTSR1 had a significantly worse prognosis than those with low NTSR1 expression (ten years survival rate of 66.2% versus 96.5%; p = 0.01). Kaplan-Meier survival graph up-to 10 years, and number of patients at risk during this period of time are shown in figure 3. Multivariate analysis with a Cox model adjusted for major prognosis risk factors, age, tumor size, SBR grade, positive ER status and lymph nodes, showed that high NTSR1 expression remained an independent prognosis marker. The relative risk of dying in women with expression of NTSR1≥80% compared to women with expression of NTSR1<80% was significantly increased (RR = 5.29, 95% confidence interval [1.04–26.88], p = 0.044).

**Table 3:** Prognosis factors and deaths stratified by NT-1 receptor expression in the invasive component of IDCs.

	NT1 receptor		
	n = 106		
	<80%	>80%	P
	n = 68	n = 38	
Positive estrogen receptor [n/number of cases studied]	46/64	23/36	NS
Positive progesterone receptor [n/number of cases studied]	46/63	25/36	NS
Tumor size (cm) [number of cases studied]	68	38	
[mean (SD)	2.08±1.35	2.71±1.4	0.007
**SBR grade** [number of cases studied]	68	38	
1	26	7	
2	31	18	
3	11	13	0.036
Number of invaded nodes[number of cases studied]	66	38	
[mean±SD]	0.86±1.7	2.11±3.4	0.05
Recurrence during follow-up [n/number of cases studied]	13/67	13/38	0.09
Deaths during follow-up [n/number of cases studied]	2/68	9/38	0.0025
**Adjuvant therapy** (102 cases studied)	65	37	
Radiotherapy [n]	61	35	NS
Chemotherapy [n]	17	19	0.01
Tamoxifen use [n]	49	29	NS
**NT** [n/number of cases studied]	28/65	20/37	NS

### NTSR1 paracrine regulation

Within the 48 patients expressing NTS in the invasive component, 20 (42%) exhibited high expression of NTSR1 (≥80%), corresponding to 20% of the whole population. Examining adjacent tissue sections of these patients, a clear regional co-localization of the ligand and its receptor was detected in all IDCs (Figure 2C). Within the population co-expressing NTS and NTSR1 the distribution among the SBR grades was altered as compared to the total population, with few patients in the grade 1 and most patients in the grade 3 (p<0.05). The size of the tumor, the recurrence and the number of death were higher in this subpopulation than in the total population. In addition, the ER alpha receptor positivity, characterized as a differentiated and good prognosis marker, was correlated with the NTS and low NTSR1 expression (p<0.05) (Table 4).

**Table 4:** Correlation of the subpopulation co-expressing NT and NT1 receptor with the major prognosis factors.

	NT invasive component		
	n = 48		
	NT-1 receptor	NT-1 receptor	P
	<80%	>80%	
	n = 28	n = 20	
Positive estrogen receptor [n/number of cases studied ]	24/27	12/20	0.049
Tumor size (cm) [mean±SD]	28	20	
[number of cases studied]	2.08±1.15	2.37±1.16	
**SBR grade** [number of cases studied]	28	20	
1	14	3	
2	12	10	
3	2	7	0.011

### NTSR1 and NTS gene expression in breast cancer microarray studies

We sought to compare our findings with those from publically available breast gene array analysis. A correlation between up regulated expression of NTSR1 with the higher grades was found studying 55 and 125 breast carcinomas, from the Ginestier and Sotiriou databases (p = 0.028 and 0.04), respectively [20], [21]. In the Chin gene profile, containing 118 frozen primary breast carcinomas, NTSR1 was found over expressed in stage IV carcinomas as compared to stage I with p = 0.003. In the same study, a correlation between NTSR1 over expression and the positive lymph node status was found (p = 0.04) [22]. No correlation was detected in the available databases between the over expression of NTSR1 and 5-year survival, noting that data were not available for longer time periods. The latter result is unsurprising, because of the very low number of deaths registered in these small populations during a survival time frame which is shorter than that observed in breast cancer patients managed with the currently available multimodality treatments. In our study the correlation between survival and NTSR1 expression was strongest at the 10-year follow-up (p = 0.01) and decreased with shortening of it, arriving at a value of 0.052 (NS) at the 5-year follow-up. Of note, studies using other parameters confirm that the NTSR1 expression is a poor prognosis marker. in the “Ma” database studying 54 patients with breast carcinomas, NTSR1 is correlated with the recurrence at 5 years (p = 0.04) [23]. In Chang's study, dealing with 24 breast ductal carcinomas classified according to the docetaxel response, NTSR1 was more intensively expressed in the group resistant to this chemotherapy agent [24].

A high correlation was found between NTS and estrogen receptor expression in the Sotiriou and Chin gene arrays ( p = 7.9 E-5 and 0.002, respectively [21], [22]). In the Chin gene array, NTS expression was also correlated with progesterone receptor expression (p = 0.003). As in our study, no other correlation was detected. The results reported here and those from the gene array profiles lead, therefore, to similar conclusions.

## Discussion

This paper evaluates the status of NTSR1 as a contributor in human breast cancer progression. One approach to address this question is to determine if paracrine NTS regulation is associated with the patients' poor outcome. We suspected that the contribution of NTSR1 in tumorigenesis occurred from local and sustained activation of the receptor rather than from circulating NTS, because NTS is a highly degradable peptide and its blood concentration rapidly drops after its release. It has been demonstrated that sustained activation of NTSR1 results in persistent NTSR1 recycling as well as signalization activation, including ERK1/2 [19], and causes sustained gene activation of MMP9 and Bcl-2 [7], [8]. In human tumor these conditions would be satisfied if NTS is synthesized and released within the vicinity of NTSR1 expressing cells. NTSR1 expression is an early event of cell transformation, because of the resulting NTSR1 promoter activation by the Wnt/β-catenin pathway [25]. Here we showed that NTSR1 is highly expressed (≥80%) in 35% of the patients with a granular labeling mostly cytosolic suggesting an intense receptor endocytosis.

The data concerning NTS expression in human cancer are sparse. Hypomethylation, or NTS regulation by Ras or Src oncogenes were described as possible mechanisms leading to expression of NTS gene in cancer [10]. Hormonal regulation was also described more specially in specific areas of the hypothalamus, and in the preoptic area, where NTS mRNA is stimulated by estrogen [26]. This effect is transcriptional and involves cAMP/protein kinase A-dependent signaling mechanisms [16]. In this paper we observed the expression of NTS and NTS mRNA in HBEC and demonstrated that NTS is an estrogen target gene in those cells. We also observed expression of NTS in tumor cells of the ductal and invasive components in breast IDCs, with a strong statistical correlation of NTS expression in both components. This latter finding, with a similar NTS repartition within the low or high NTSR1 expressing patients, suggests that NTS gene remains constitutively expressed during the neoplastic process, rather than being deregulated. In parallel, we observed a frequent regional co-localization of both markers in adjacent tissue sections from the same tumor, suggesting NTSR1 activation. Together with the expression of NTSR1, these data validate our hypothesis of the NTS paracrine regulation of transformed epithelial cells during the neoplastic process, with NTS released from the surrounding normal breast tissue or from the breast tumor.

High NTSR1 expression was significantly associated with the SBR grade, the size of the tumor, and the number of metastatic lymph nodes, and ultimately with death of the patients. These findings support the deleterious effects of NTS found in breast cancer cells [7]. NTS and NTSR1 are implicated in several detrimental functions linked to the neoplastic progression, including proliferation of the pancreas, prostate, colon and lung cancer cells [6], protection of breast cancer cells against apoptosis [8], and induction of the proinvasive potential of colon cancer cells [25]. More recently, it was shown that NTSR1 activation results in EGFR transactivation by the shedding of transforming growth factor-α (TGF-α) in pancreas, and HB-EGF or amphiregulin in prostate cancer cells, both leading to ERK1/2 activation [27], but also EGFR expression [28]. These findings point out that the poor prognosis attributed to patients with highly NTSR1 expressing IDCs may be directly related to the expression of its natural ligand NTS, with the continuous activation of the NTSR1, leading to enhanced cancer cell survival, invasiveness potential, and metastasis [7], [8].

In conclusion, based on a series of 106 patients with invasive ductal breast cancer, we provide evidence for NTS/NTSR1 as a contributor to breast cancer progression. Identification of breast cancer patients characterized by paracrine NTS/NTSR1 signaling pathway, as evidenced in the present study, will provide alternative strategies to improve the treatment of IDCs. These findings support the therapeutic potential of NTS/NTSR1 inhibition or drug cellular targeting through NTSR1 in advanced stages of human breast cancers.

## Materials and Methods

### Breast Biopsies

Clinical file of 106 patients completely resected for invasive ductal breast cancers (IDCs) by tumorectomy or mastectomy, at the Gynecology Department, Hôtel-Dieu Hospital, Paris, were studied. Patients were diagnosed by the same oncologist (Dr Y. Decroix) for a period from June 1984 through May 1998. Clinico-pathological information was derived retrospectively from patient records. Survival and follow-up durations were measured as the time between the first histological confirmation of breast cancer and the last consultation in the department, or death. Patient records were reviewed retrospectively for demographical characteristics, clinical data, outcome, and survival. The histological diagnosis was routinely checked by microscopic examination of sections stained with hematoxylin-eosin.

### Ethics

The following studies were conducted on tissues obtained from patients between 1984 and 1998. The experiments reported here were carried out under the current French ethical regulations as defined by the Huriet-Sérusclat act of 12/20/1988. Under this act, institutional review board approval was not required. Accordingly, patients were specifically asked for a verbal informed consent only, and consequently no IRB number approval was requested. The study was carried out according to the Declaration of Helsinki principles and in agreement with the French laws on biomedical research.

### Immunohistochemistry

Breast tumor sections of 5 µm thickness were analyzed by immunohistochemistry for NTSR1, NTS, ER, and PR staining, using the following antibodies: a NTSR1 goat polyclonal antibody (C-20 Santa Cruz USA), a NTS rabbit antibody (NA1230 Biomol, USA), a ER-α monoclonal antibody (Santa Cruz), a PR monoclonal antibody (Santa Cruz). Immunostaining was carried out on deparaffinized sections using the streptavidin biotin peroxidase complex method as described previously by Souazé et al [7]. All slides were counterstained with hematoxylin. A semi-quantitative estimation of the number of positive cells was performed by counting 1 000 reactive and non-reactive cells in ten successive fields at the original 200× magnification.

### Normal breast tissues

Normal human breast epithelial cells (HBEC) were cultured as previously described in Gompel et al. [29]. Additional biopsies of normal breast tissues from 25 premenopausal women of various ages (18–50 years) undergoing plastic surgery were obtained according to the French regulations on clinical experimentation.

### RNA extraction and RT-PCR

The protocols for total RNA extraction, reverse-transcription reaction (RT), and PCR are documented in Souazé et al [30]. RT was performed on 1 µg of total RNA using a specific NTSR1 primer (5′-GCTGACGTAGAAGAG-3′) or 50 pmol of oligo dT and oligo dN. The PCR amplification was performed on a 1∶5 v/v of the RT reaction using 25 pmol of each primer 5′-CGTGGAGCTGTACAACTTCA-3′ and 5′-CAGCCAGCAGACCACAAAGG-3′ for NT1 receptor, and 5′-AAGCACATGTTCCCTCTT-3′ and 5′-CATACAGCTGCCGTTTCAGA-3′ for NTS, and 1 unit of Taq polymerase (Applied Biosystems, Courtaboeuf, France). The amplification profile consisted of denaturation at 94°C for 30 s, annealing at 57°C for 45 s, and extension at 72°C for 45 s. The PCR cycle were preceded by denaturation at 95°C for 15 min and were followed by a final extension at 72°C for 7 min.

### Hormonal treatments

HBEC were synchronized for 40 h in Ham F10 phenol red free medium containing 20 µM lovastatin. Synchronization was stopped by adding 2 mM mevalonate to the hormone-containing medium. Subsequently, cells were treated 48 h in a phenol red free medium containing 5% of compatible human serum with 10 nM estradiol (E2) with or without 1 µM ICI 182780.

### Statistics

Analyses were processed with StatView Version 5 (Abacus Concepts, Berkeley, CA., USA). Descriptive statistics were performed for each variable; quantitative results are presented as mean±SD and qualitative results are presented as a distribution of a number of patients. To compare the two groups, χs^2^ test was used for qualitative variables and t test for quantitative variables. A p value<0.05 was accepted as significant. Survival analysis was performed by Kaplan-Meier method and comparison with Log-Rank test. For multivariate analysis, Cox model was performed using R statistical package.
